# Comparative Genome Analyses of *Clavibacter michiganensis* Type Strain LMG7333^T^ Reveal Distinct Gene Contents in Plasmids From Other *Clavibacter* Species

**DOI:** 10.3389/fmicb.2021.793345

**Published:** 2022-02-01

**Authors:** Eom-Ji Oh, In Sun Hwang, In Woong Park, Chang-Sik Oh

**Affiliations:** ^1^Department of Horticultural Biotechnology, College of Life Science, Kyung Hee University, Yongin, South Korea; ^2^Graduate School of Biotechnology, Kyung Hee University, Yongin, South Korea

**Keywords:** *Clavibacter michiganensis*, comparative genomics, complete genome sequence, Gram-positive bacteria, type strain

## Abstract

*Clavibacter michiganensis*, a Gram-positive, plant-pathogenic bacterium belonging to Actinobacteria, is a causal agent of bacterial canker in tomatoes. Although LMG7333^T^ is the type strain of *C*. *michiganensis*, it has not been used in many studies, probably because of a lack of the complete genome sequence being available. Therefore, in this study, the complete genome sequence of this type strain was obtained, and comparative genome analysis was conducted with the genome sequences of two other *C. michiganensis* strains and type strains of *Clavibacter* species, of which their complete genome sequences are available. *C*. *michiganensis* LMG7333^T^ carries one chromosome and two plasmids, pCM1 and pCM2, like two other *C. michiganensis* strains. All three chromosomal DNA sequences were almost identical. However, the DNA sequences of two plasmids of LMG7333^T^ are similar to those of UF1, but different from those of NCPPB382, indicating that both plasmids carry distinct gene content among *C. michiganensis* strains. Moreover, 216 protein-coding sequences (CDSs) were only present in the LMG7333^T^ genome compared with type strains of other *Clavibacter* species. Among these 216 CDSs, approximately 83% were in the chromosome, whereas others were in both plasmids (more than 6% in pCM1 and 11% in pCM2). However, the ratio of unique CDSs of the total CDSs in both plasmids were approximately 38% in pCM1 and 30% in pCM2, indicating that the high gene content percentage in both plasmids of *C. michiganensis* are different from those of other *Clavibacter* species, and plasmid DNAs might be derived from different origins. A virulence assay with *C. michiganensis* LMG7333^T^ using three different inoculation methods, root-dipping, leaf-clipping, and stem injection, resulted in typical disease symptoms, including wilting and canker in tomato. Altogether, our results indicate that two plasmids of *C. michiganensis* carry distinct gene content, and the genome information of the type strain LMG7333^T^ will help to understand the genetic diversity of the two plasmids of *Clavibacter* species, including *C. michiganensis*.

## Introduction

Plant pathogenic bacteria belonging to the genus *Clavibacter* are Gram-positive and often cause severe damage to crops (Eichenlaub and Gartemann, 2011). This genus was reclassified from *Corynebacterium* and newly identified as a new genus, including several phytopathogenic bacteria (Davis et al., 1984). The genus *Clavibacter* contained the single species, *C*. *michiganensis* with nine subspecies, according to its host specificity (Eichenlaub et al., 2007; Gonzalez and Trapiello, 2014; Yasuhara-Bell and Alvarez, 2015; Oh et al., 2016). Recently, seven out of eight subspecies except *C*. *michiganensis* subsp. *chilensis* (non-pathogenic bacterium) were reclassified as separate species based on the genomic analysis in addition to its host specificity (Li et al., 2018; Méndez et al., 2020): *C*. *michiganensis* (bacterial canker and wilting in tomato) (Smith, 1910), *C*. *sepedonicus* (ring rot in potato) (Manzer and Genereux, 1981), *C*. *nebraskensis* (Goss’s leaf blight and wilting in maize) (Vidaver and Mandel, 1974), *C*. *insidiosus* (bacterial wilt in alfalfa) (McCulloch, 1925), *C*. *tessellarius* (leaf freckles and leaf spots in wheat) (Carlson and Vidaver, 1982), *C*. *phaseoli* (bacterial leaf yellowing in beans) (Gonzalez and Trapiello, 2014), and *C*. *capsici* (bacterial canker in pepper) (Oh et al., 2016) and a non-pathogenic *C*. *californiensis* (Yasuhara-Bell and Alvarez, 2015). Although Méndez et al. (2020) also propose reclassifying *C*. *michiganensis* subsp. *chilensis* to *C*. *phaseoli*, we kept the original subspecies name to distinguish this strain from the original *C*. *phaseoli* in this study.

Disease through *Clavibacter* bacterial infection resulted in economic damage due to the significant yield loss. For example, *C*. *michiganensis* was first reported in 1910 in North America (Smith, 1910), and since then, this pathogen has caused significant economic loss (Xu et al., 2012; Peritore-Galve et al., 2020). Additionally, *C*. *sepedonicus* was designated a quarantine pathogen in Europe owing to its severe economic damage annually (Van der Wolf et al., 2005). *C*. *michiganensis* has been studied relatively well among *Clavibacter* species. Although virulence mechanisms of this pathogen are not well known yet, previous research shows that two plasmid-borne genes, *celA* and *pat-1*, play essential roles in *C*. *michiganensis* pathogenicity (Dreier et al., 1997; Jahr et al., 2000; Burger et al., 2005; Hwang et al., 2019). Moreover, a pathogenicity island (PAI) region, which contains putative virulence genes such as the *ppa*, *chp*, and *pel* gene families, was found in the chromosome (Gartemann et al., 2008; Stork et al., 2008). In tomato seeds, *C*. *michiganensis* ssp. *chilensis* and *C*. *californiensis* were isolated and exhibited non-pathogenic effects to tomato plants (Yasuhara-Bell and Alvarez, 2015). Notably, these two non-pathogenic bacteria missed most virulence genes and/or putative virulence genes possessed by pathogenic *C*. *michiganensis* (Méndez et al., 2020).

So far, the complete genome sequences of type strains of only three *Clavibacter* species have been reported: *C*. *sepedonicus* ATCC33113^T^ (Bentley et al., 2008), *C*. *nebraskensis* NCPPB2581^T^ (GenBank accession #: HE614873), and *C*. *capsici* PF008^T^ (Bae et al., 2015). In the case of *C*. *insidiosus* species, the complete genome sequence of strain R1-1, which is not a type strain, is available in the public database (Lu et al., 2015). Based on these whole genome sequences of *Clavibacter* species in the public database, studies on *Clavibacter* species at the genomic level have recently increased. Moreover, newly sequenced genome data of *Clavibacter* strains have allowed us to conduct comparative genome analysis at the species or strain level (Tambong, 2017; Osdaghi et al., 2020). Indeed, several comparative genomic approaches have been conducted to target *Clavibacter* species isolated from specific geographic regions using genome database (Thapa et al., 2017; Hwang et al., 2020a; Méndez et al., 2020), and the genomic analysis is becoming more important to the genus *Clavibacter* research.

In the case of a tomato pathogen *C. michiganensis* species, only four strains, NCPPB382 (Gartemann et al., 2008), UF1, and two Chile isolates, VL527 and MSF322 (Méndez et al., 2020), were completely sequenced. Most of *C*. *michiganensis* genome sequences have not been completely assembled (Thapa et al., 2017). Although the draft sequence of *C*. *michiganensis* type strain LMG7333^T^ (= NCPPB 2979^T^, CFBP 2352^T^, DSM 46364^T^) was already reported, it has not yet been completed (Li and Yuan, 2017; Li et al., 2018). The draft genome sequencing of *C*. *michiganensis* LMG7333^T^ was conducted previously by PacBio-SMRT sequencing technology (Li and Yuan, 2017), resulting in five contigs. Because this genome sequence was not complete, there was a limitation to use it as a reference genome of *C*. *michiganensis* species. For these reasons, NCPPB382 rather than a type strain of *C*. *michiganensis* was mainly used for many studies that have focused on molecular mechanisms to understand virulence mechanism(s) even though this type strain can induce typical disease as severe as *C*. *michiganensis* NCPPB382 on tomato plants (Hwang et al., 2019).

In this study, we report the completed genome sequence of *C*. *michiganensis* type strain LMG7333^T^ and use this sequence for comparison with other previously published genomes of *C*. *michiganensis* to obtain more valid insights onto genetic diversity among pathogenic *Clavibacter* species. Additionally, we conducted a virulence assay with this type strain using three inoculation methods. Our results suggest that comparative genome analysis with the complete genome sequence of *C*. *michiganensis* type strains LMG7333^T^ can provide a genomic resource to understand the genetic diversity among *Clavibacter* species better.

## Materials and Methods

### Genome Sequencing, Assembly, and Annotation of *Clavibacter michiganensis* LMG7333^T^

The genomic DNA (gDNA) of *C*. *michiganensis* LMG7333^T^ was extracted using the solution-based HiGene Genomic DNA Prep kit (BIOFACT, Daejeon, Korea), according to the manufacturer’s instructions. Using extracted gDNA, whole genome sequencing of LMG7333^T^ was conducted using combined methods of two sequencing platforms, PacBio RSII and Illumina HiSeqXten (Macrogen, Seoul, Korea), for genome sequence completion. The reads obtained from PacBio RSII were assembled by Hierarchical Genome Assembly Process (Chin et al., 2013). HiseqXten reads were also used to correct assembly by Pilon v1.21 (Walker et al., 2014). Based on the assembled sequences, gene prediction and annotation were conducted by Prokka v1.13 (Seemann, 2014). Then, InterProScan v5.30–69.0 (Jones et al., 2014) and psiblast v2.4.0 (Camacho et al., 2009) with EggNOG DB v4.5 (Huerta-Cepas et al., 2016) and the Clustering Orthologous Groups (COG) database v2014 (Tatusov et al., 2000) were used to confirm the predicted proteins. Circos program v0.69.3 (Krzywinski et al., 2009) was used for drawing circular maps of the genome.

### Genome Comparison of *Clavibacter michiganensis* LMG7333^T^

The completed whole genome sequence of *C. michiganensis* LMG7333^T^ type strain was first compared by aligning the EasyFig program (Sullivan et al., 2011) and NCBI blast program with those of *C. michiganensis* NCPPB382 (GenBank accession #: AM711865, AM711866, and AM711867) (Gartemann et al., 2008) and UF1 (GenBank accession #: CP033724, CP033725, and CP033726). Then, it was also compared with completed genome sequences of nine other *Clavibacter* species, including several type strains such as *C*. *sepedonicus* ATCC33113^T^ (GenBank accession #: AM849034) (Bentley et al., 2008), *C*. *capsici* PF008^T^ (GenBank accession #: CP012573, CP012574, and CP012575) (Bae et al., 2015), *C*. *insidiosus* R1-1 (GenBank accession #: CP011043, CP011044, CP011045, and CP011046) (Lu et al., 2015), *C*. *nebraskensis* NCPPB2581^T^ (GenBank accession #: HE614873), *C*. *insidiosus* LMG3663^T^ (GenBank accession #: MZMO01000001, MZMO01000002, and MZMO01000003), *C*. *tessellarius* ATCC33566^T^ (GenBank accession #: MZMQ01000001 and MZMQ01000002) (Li and Yuan, 2017; Li et al., 2018), *C. phaseoli* CFBP8627^T^ (GenBank accession #: QWGV00000000), *C. michiganensis* subsp. *chilensis* CFBP8217^T^ (GenBank accession #: QWGS01000000), and *C*. *californiensis* CFBP8216^T^ (GenBank accession #: QWEE01000000) (Méndez et al., 2020). The genome sequence of *Leifsonia xyli* subsp. *cydontis* DSM46306^T^ (GenBank accession #: CP006734) (Monteiro-Vitorello et al., 2013), which belongs to the same family as genus *Clavibacter*, was used as an out group. To understand the phylogenetic relationships among the analyzed strains, OrthoANI values were calculated, and UPGMA dendrograms were generated using the Orthologous ANI Tool of ChunLab (Lee et al., 2016). Moreover, the pan-genome analysis was conducted, and results were shown through a Venn diagram (Bardou et al., 2014).

### Virulence Assay in Tomato Plants

*C. michiganensis* strain LMG7333^T^ was grown at 26°C in KB medium (20 g proteose peptone No. 3, 1.5 g K_2_HPO_4_, 6 mL 1 M MgSO_4_, 16 mL 50% glycerol, and 15 g agar in the total volume of 1 L) for 3 days and inoculated into tomato plants using three different inoculation methods, which are leaf-clipping inoculation, stem injection, and root-dipping inoculation. Ten-day-old and 3- and 4-week-old tomato plants (*Solanum lycopersicum* L. cv. “Betatini”) were used for the root-dipping inoculation, leaf-clipping inoculation, and stem injection, respectively. For the leaf-clipping method, the bacterial inoculum was prepared and adjusted to a concentration of 2 × 10^8^ CFU/mL (OD_600_ = 0.4). Sterilized scissors were dipped into this inoculum, and then half of the leaves were cut using scissors (Hwang et al., 2020b). Three leaves located in different directions of each plant and at least five plants were used for each treatment. For stem injection, a bacterial concentration was adjusted to 10^9^ CFU/mL (OD_600_ = 2.0), and the 20 μL bacterial suspension was injected into tomato stems above 2 cm from the cotyledon after making a hole with a needle. At least four plants were used for each treatment. For the root-dipping inoculation method, 10-day-old seedlings were pulled out from soil, and their roots were dipped in 1 mL of bacterial suspension (10^9^ CFU/mL) for 30 min. The seedlings were then replanted into pots. Five plants were used for each treatment. *C. michiganensis* LMG7333^T^ was resuspended in sterilized 10 mM MgCl_2_ buffer, and this buffer was used as a negative control in all inoculation methods.

### Bacterial Growth Measurement *in planta*

After inoculation with *C*. *michiganensis* LMG7333^T^ by stem injection, tomato stems were collected at 0, 7, 14, 21, and 28 days after inoculation (dai). For sample collection, 3 cm lengths of inoculated stems were harvested from four different sites, which are the inoculated site and 10, 15, and 20 cm above from the inoculated site. Then, 2 mL of 10 mM MgCl_2_ was added to ground samples and the supernatant was dotted onto KB media after 10-fold serial dilution. Three plants were used for each repetition, and experiments were repeated at least three times.

### Statistical Analysis

For statistical analysis of bacterial growth of *Clavibacter michiganensis* LMG7333^T^ *in planta*, Duncan’s multiple range test was conducted (*p* < 0.05).

## Results

### Complete Genome Information of *Clavibacter michiganensis* LMG7333^T^

**FIGURE 1:**
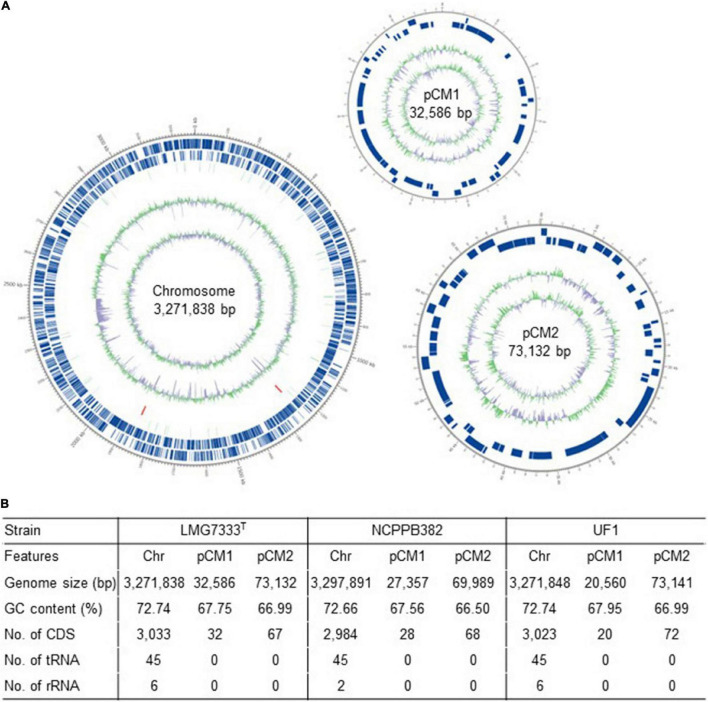
Genome information of *C*. *michiganensis* type strain LMG7333^T^. **(A)** A circular map drawn by Circos program. Circles outside to the center are represented as CDS on the forward strand, CDS on the reverse strand, tRNA, rRNA, GC content, and GC skew. **(B)** Genome summary of three *C*. *michiganensis* strains, LMG7333^T^, NCPPB382, and UF1. Chr, chromosome; pCM1 and pCM2, plasmids; CDS, coding sequences.

**FIGURE 2:**
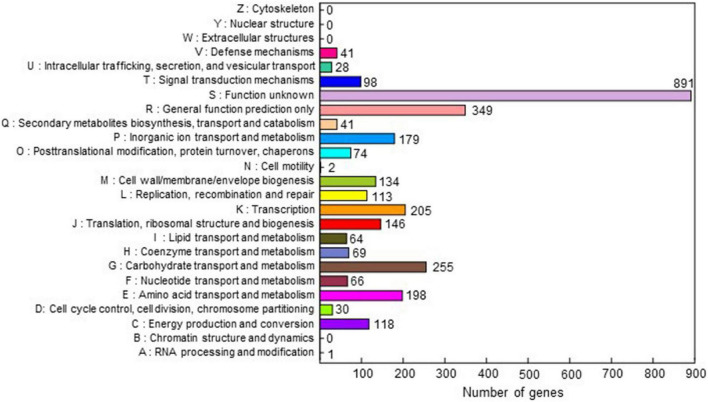
Clusters of orthologous groups (COG) of the annotated CDSs of *C*. *michiganensis* type strain LMG7333^T^. COG database v2014 was used for analysis. Annotated genes were classified into 21 categories of 25. The *X*-axis represents the number of genes, and the *Y*-axis indicates each COG cluster. The numbers next to each bar indicate the number of genes in each COG cluster.

The complete genome sequence of *C*. *michiganensis* LMG7333^T^ was obtained through the combination of PacBio RSII and Illumina HiSeqXten sequencing methods, and the genome coverage was 320X. From HiSeqXten, we got 9,154,590 reads, and after assembly, the N50 value was 3,271,838. Finally, we obtained three contigs, one circular chromosome (GenBank accession #: CP080437), and two plasmids, pCM1 and pCM2 (GenBank accession #: CP080438 and CP080439). Their genome sizes and G + C contents are 3.27 Mb with 72.74% G + C content, 32 kb with 67.75% G + C content, and 73 kb with 66.99% G + C content, respectively (Figures 1A,B). This genomic composition of *C*. *michiganensis* LMG7333^T^ is similar to those of NCPPB382 and UF1 strains, of which the complete genome sequences are available. However, their sizes are different from one another, particularly, plasmid sizes (Figure 1B). Genome annotation revealed 3,033 protein-coding sequences (CDSs) as well as 45 tRNAs and 6 rRNAs in the chromosome and 32 and 67 CDSs in pCM1 and pCM2, respectively (Figure 1B). The annotated proteins were categorized into 21 COG. Approximately 40% of CDSs were categorized into two groups for the function unknown (S; 891 CDSs) and the general function only (R; 349 CDSs), which are poorly characterized, in the COG database (Figure 2). COG groups for carbohydrate transport and metabolism (G; 255 CDSs); transcription (K; 205 CDSs); amino acid transport and metabolism (E; 198 CDSs); inorganic ion transport and metabolism (P; 179 CDSs); translation, ribosomal structure, and biogenesis (J; 146 CDSs); and cell wall/membrane/envelope biogenesis (M; 137 CDSs) were found to be dominant groups. In contrast, only two CDSs were categorized into cell motility groups (N) (Figure 2). Some CDSs were not grouped into specific COG or grouped to two or more COG groups. There are no CDSs for flagella formation, which is consistent with a previous study reporting that *C*. *michiganensis* LMG7333^T^ did not have flagella (Oh et al., 2016).

### Genome Comparison Among Complete Genome Sequences of Three *Clavibacter michiganensis* Strains

**FIGURE 3:**
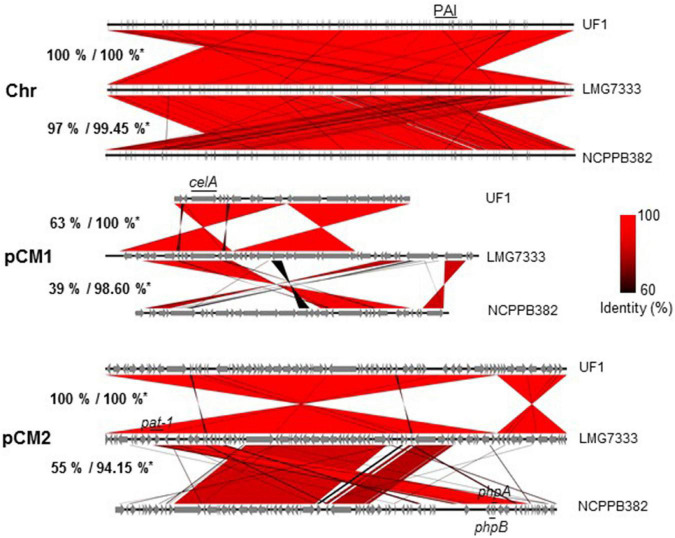
Genome comparison of three *C*. *michiganensis* strains. Genome alignment of three *C*. *michiganensis* strains. *Percentages indicate represented query cover/identity of a chromosome (Chr) and two plasmids, pCM1 and pCM2, of *C*. *michiganensis* NCPPB382 and UF1 based on *C*. *michiganensis* LMG7333^T^. The identical parts were aligned in red, and the key virulence genes such as *celA* and *pat-1* genes or a pathogenicity island (PAI) are shown.

As shown in Figure 1B, three *C*. *michiganensis* strains differed in the sizes of a chromosome and two plasmids. Here, three genome sequences were compared to examine how different they were. First, the sequence identity of chromosomal DNA among the three strains was almost 100% identical although the query cover between LMG7333^T^ and NCPPB382 strains was 97% (Figure 3). As with other *C. michiganensis* strains, *C*. *michiganensis* LMG7333^T^ has about 120 kb of pathogenicity island (PAI) region from 2,334,274 to 2,464,147 bp on its chromosome (Figure 3 and Supplementary Figure 1). In the *chp* region of PAI, the putative virulence genes, such as the *chp*, *ppa*, and *pel* gene families, were intact like those in the NCPPB382 strain. Moreover, the *tomA* gene was found in the *tomA* region of PAI (Supplementary Figure 1). Two other important virulence genes, *celA*, and *pat-1* genes are located from 7,321 to 9,561 bp in pCM1 and from 7,833 to 8,675 bp in pCM2 of *C. michiganensis* LMG7333^T^ (Figure 3). The presence of an intact PAI region, *celA* and *pat-1* genes, suggests that *C*. *michiganensis* LMG7333^T^ can also infect tomato plants to cause typical disease symptoms like the NCPPB382 stain.

The size and sequence query cover of the pCM1 plasmid of LMG7333^T^ was the most different from those of two other strains although the sequence identity in the matched regions was almost 100% identical. Its size was the biggest among the three strains, and the matched regions were only 63% with UF1 pCM1 and only 39% with NCPPB382 pCM1 (Figure 3). Among unmatched regions in pCM1 of LMG7333^T^, some regions were unique to this strain, whereas others were only matched with that of NCPPB382. Most of the genes in these unmatched regions are annotated as hypothetical proteins, indicating that the origin and functions of these genes are unknown. In the public database, *C*. *michiganensis* NCPPB382 pCM1 has genes for cellulase (*celA*), portioning protein (*parA* and *parB*), and replication origin (*repA*) and several hypothetical proteins. When the sequence of pCM1 was compared with these annotated genes of pCM1 in *C*. *michiganensis* NCPPB382, we found that *C*. *michiganensis* LMG7333^T^ pCM1 also contained these annotated genes except the *traG* gene encoding putative conjugal transfer protein (Załuga et al., 2014). Gene annotation also revealed that the *espI* gene, which is a component of the type VII ESX-1 secretion system (Zhang et al., 2014), was found in the pCM1 of LMG7333^T^ but not NCPPB382, whereas both pCM2 plasmids have the *espI* homologous gene.

The size and sequence of LMG7333^T^ pCM2 plasmid were almost 100% identical to that of the UF1 strain, whereas the matched regions with NCPPB382 pCM2 were only approximately 37 kb of 73 kb, which is only 55% of the total plasmid size with 91–99% sequence identity (Figure 3). In the matched region of pCM2 plasmids, all three *C*. *michiganensis* strains possess *pat-1* genes, encoding a putative serine protease, which was known as a plasmid-borne virulence factor of *C*. *michiganensis* (Burger et al., 2005). In the case of *C. michiganensis* NCPPB382, there are more *pat-1* paralogs, *phpA* and *phpB*, in pCM2. However, *C. michiganensis* LMG7333^T^ lacks these two genes. The unmatched region covered by 45% of the total plasmid size was unique to each strain.

Overall, these results indicate that, although three *C*. *michiganensis* strains have similar genome size and major virulence factors, like PAI, *celA*, and *pat-1*, in their genomes, the plasmid sequences are varied, and both pCM1 and pCM2 of LMG7333^T^ contain unique DNA sequences mostly annotated as hypothetical proteins. Moreover, genome comparison among three *C*. *michiganensis* strains using complete genome sequences indicates that *C*. *michiganensis* LMG7333^T^ genome sequence can be used as another reference genome of *C*. *michiganensis*.

### Genome Comparison of *Clavibacter michiganensis* LMG7333^T^ With Other *Clavibacter* Species

**FIGURE 4:**
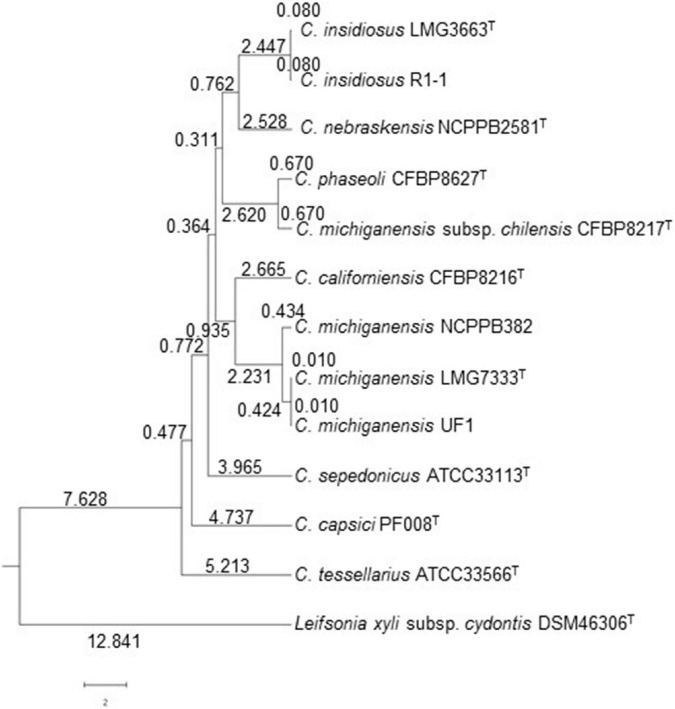
Phylogenetic tree of *C*. *michiganensis* type strain LMG7333^T^ with other *Clavibacter* species and subspecies based on ANI. This analysis was conducted using OrthoANI software. *Leifsonia xyli* subsp. *cydontis* DSM46306^T^ was used for outgroup. Numbers indicate genetic similarity (GS) coefficient value. T, type strain.

Based on genomic information, average nucleotide identity (ANI) values were calculated among *C*. *michiganensis* LMG7333^T^ and nine other *Clavibacter* bacteria, including type strains of other *Clavibacter* species and *Leifsonia xyli* subsp. *cydontis* as an outgroup to measure the genetic distance among genomes. Based on ANI data, three *C. michiganensis* strains were clustered into the same clade with more than 99% ANI value, and non-pathogenic *Clavibacter californiensis* was the closest to *C*. *michiganensis* with almost 95% ANI value (Figure 4 and Supplementary Table 1). There was the second clade, including *C*. *insidiosus*, *C*. *nebraskensis*, *C*. *phaseoli*, and *C*. *michiganensis* subsp. *chilensis*, which is close to the clade of *C*. *michiganensis*. Type strains of *C*. *capsici* PF008^T^, *C*. *sepedonicus* ATCC33113^T^, and *C*. *tessellarius* ATCC33566^T^ are phylogenetically distinct from the *C*. *michiganensis* clade.

**FIGURE 5:**
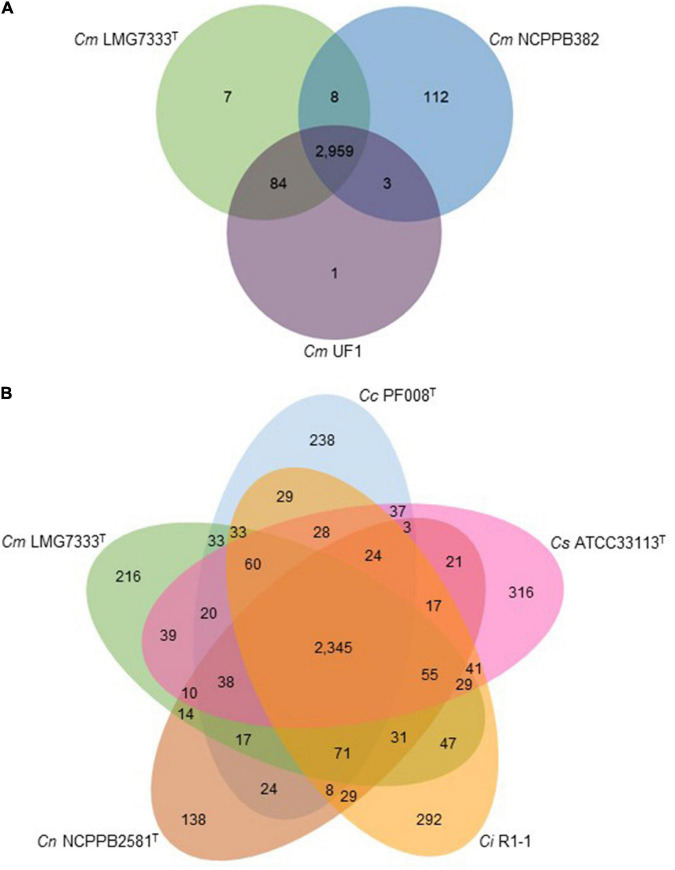
Comparative genome analysis of *C*. *michiganensis* LMG7333^T^ and other *Clavibacter* strains. **(A)** Venn diagram of three *C*. *michiganensis* strains. **(B)** Venn diagram of *C*. *michiganensis* LMG7333^T^ and four other type strains of *Clavibacter* species. Numbers represent gene numbers, which are common or unique to each strain. *Cm*, *C*. *michiganensis*; *Cc*, *C*. *capsici*; *Cs*, *C*. *sepedonicus*; *Ci*, *C*. *insidiosus*; *Cn*, *C*. *nebraskensis*.

Using genome sequences of pathogenic *Clavibacter* strains, pan-genome analysis was also conducted. Three *C. michiganensis* strains were first compared, and they have 2,959 common genes. *C. michiganensis* LMG7333^T^ only has seven unique genes in its genome. Moreover, it has 8 and 84 genes overlapped with *C. michiganensis* NCPPB382 and UF1, respectively (Figure 5A). Among the seven unique genes in *C. michiganensis* LMG7333^T^, only one gene was annotated as RNA helicase, and the other six genes were annotated as hypothetical proteins. The specific genes in other two *C. michiganensis* strains were also annotated as hypothetical proteins. *C*. *michiganensis* LMG7333^T^ has 91 unique genes compared with *C*. *michiganensis* NCPPB382. Among these genes, 50 genes are located in the chromosome of the type strain, and the other 41 genes exist in two plasmids, 17 and 24 genes in pCM1 and pCM2, respectively. Among these 91 genes, only 17 genes were annotated (Supplementary Table 2).

BLAST analysis with 91 genes unique to *C*. *michiganensis* LMG7333^T^ compared with *C*. *michiganensis* NCPPB382 was conducted to identify the possible origin of these genes. Based on the analysis results, these 91 genes exhibited significant homology with diverse organisms, including other *Clavibacter* species (Supplementary Table 2). One gene exists in only *C*. *michiganensis* LMG7333^T^, and nine genes are found in only two *C*. *michiganensis* strains, LMG7333^T^ and UF1. Twenty-seven genes were shared with other *C*. *michiganensis* strains, MSF322 and VL527 (Méndez et al., 2020). Twenty-two genes are shared with other *Clavibacter* bacteria, such as *C*. *insidiosus* (10), *C*. *capsici* (7), *C*. *nebraskensis* (4), and *C*. *sepedonicus* (1). The remaining 32 genes are shared with diverse organisms, 11 families, and 18 genera. Most of these genes are shared with bacteria, including four families, *Actinomycetaceae* (1), *Cellulomonadaceae* (1), *Microbacteriaceae* (18), and *Micrococcaceae* (2), which belong to the phylum *Actinobacteria*. In particular, the 18 genes shared with the *Microbacteriaceae* family, including *Clavibacter* genus, were shared with six genera, *Microbacterium* (5), *Cryobacterium* (1), *Curtobacterium* (8), *Leifsonia* (2), *Leucobacter* (1), and *Rathayibacter* (1). Finally, among 91 *C*. *michiganensis* LMG7333^T^ genes, 77 genes are found only in the *Microbacteriaceae* family. These results indicate that the *C*. *michiganensis* LMG7333^T^ might obtain most foreign genes from the same bacterial family.

The *C. michiganensis* LMG7333^T^ genome was also compared with those of four other *Clavibacter* type strains. Type strains of five *Clavibacter* species have 2,345 core genes. Interestingly, each *Clavibacter* has 138–316 specific genes in their genomes (Figure 5B). Most of these genes were annotated as hypothetical proteins and categorized mostly into unknown function genes, carbohydrate transport, and metabolism as well as transcription proteins in the COG database (Supplementary Table 3). When pan-genome analysis was conducted, duplicated genes were regarded as one gene. Therefore, the total CDS number (Figure 1B) and CDS number of pan-genome analysis (Figure 5B) were slightly different.

### Disease Development in Tomato Plants by *Clavibacter michiganensis* LMG7333^T^

**FIGURE 6:**
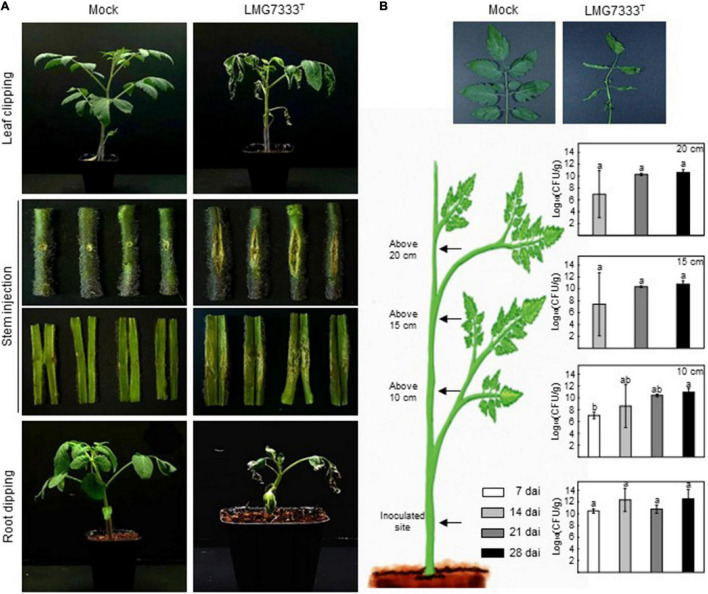
Virulence of *C*. *michiganensis* LMG7333^T^ on tomato plants. **(A)** Typical disease symptoms of *C*. *michiganensis* LMG7333^T^, according to inoculation methods. Photos were taken at 30 days after inoculation (dai) for leaf clipping and stem inoculation and for root-dipping inoculation at 10 dai. **(B)** Bacterial numbers after stem inoculation at the indicated time points. The graphs from bottom to top indicate cfu/g at the inoculation site, 10, 15, and 20 cm above site from the inoculation point. Error bars indicate standard deviation (*n* = 3). The different letters indicate statistically significant differences as determined by Duncan’s multiple range test (*p* < 0.05).

In our previous study, *C. michiganensis* LMG7333^T^ and its mutant strain were used to investigate the gene function involved in virulence (Hwang et al., 2019). For the virulence assay or mutant screening, the root-dipping method was the best way to observe typical wilting symptoms within a short period. Here, we examined the virulence of *C*. *michiganensis* LMG7333^T^ on tomato plants in various ways. Tomato leaves infected with *C*. *michiganensis* LMG7333^T^ using the leaf-clipping method exhibited wilting symptoms from the cut part of leaves (Figure 6A). As bacteria are directly inoculated into the plants, it was successfully spread along the leaf veins and induced wilting symptoms on the inoculated leaves. Eventually, this bacterium spread throughout the plant and caused typical wilting symptoms even on non-inoculated leaves and branches (Figure 6A). In contrast, no disease symptoms and no physiological disorders were found in buffer-treated tomato plants.

Stem inoculation after artificial wounding, which is a conventional method for studying *C*. *michiganensis*, induced large canker lesions at the site of inoculation (Figure 6A). The inoculated bacteria were gradually spread along the xylem toward the tip, and approximately from 21 dai, leaves on the tomato tip exhibited wilting symptoms (Figure 6B). Movement of *C*. *michiganensis* LMG7333^T^ within the stem was confirmed by bacterial growth *in planta*. From 7 dai, bacteria were detected at 10 cm above the inoculation site, and from 14 dai, it was isolated at the topper site of the stem, indicating that *C*. *michiganensis* LMG7333^T^ could move and colonize in areas higher than the inoculated site (Figure 6B). Altogether, typical bacterial cankers at the site of injection as well as wilting symptoms at the branch and leaves were observed in stem-inoculated tomato plants.

Disease symptoms through this bacterial infection were also caused by the root-dipping methods (Figure 6A). Although this method has not been widely used, disease symptoms were shown within 10 days by the root-dipping method in 8–10-day-old tomato plants. Because this pathogen can enter the tomato plant through its roots during natural infection, the virulence or pathogenicity assay in tomato seedlings by root infection may be more significant than the stem inoculation (Figure 6A). The observation that *C*. *michiganensis* LMG7333^T^ infection in tomato plants resulted in typical disease symptoms by three different inoculation methods indicates that this pathogen with the completed whole genome sequence might be valuable as a standard strain for *C*. *michiganensis* study and its interaction with the plants.

## Discussion

So far, genome sequences of many *Clavibacter* bacteria have been reported and used for understanding these bacteria at the genomic level. For *C*. *michiganensis*, the complete whole genome sequence of *C*. *michiganensis* strain NCPPB382 was first reported (Gartemann et al., 2008), and this genomic information has provided the opportunity to reveal many putative virulence genes in a PAI region, such as the *chp*, *ppa*, and *pel* gene families, and also the presence of two plasmids, pCM1 and pCM2. Since the NCPPB382 genome sequence became available, many draft genome sequences of *C*. *michiganensis* strains have been reported (Thapa et al., 2017; Méndez et al., 2020). Genome sequencing of *C*. *michiganensis* type strain *C*. *michiganensis* LMG7333^T^ was previously conducted with PacBio-SMRT sequencing technology, and its draft genome sequence was reported (Li and Yuan, 2017). However, this report showed five contigs. Because this genome sequence was not complete, there was a limitation in using it as a reference genome of *C*. *michiganensis* species to understand the genetic diversity in either chromosome or plasmids among strains of this species. In this study, for completion of genome sequencing of type strain LMG7333^T^, we combined two sequencing platforms, PacBio RSII and Illumina HiSeqXten, and could successfully obtain the complete genome sequence with three contigs, one chromosome and two plasmids (Figure 1). This could make it possible to perform comparative genomic analyses among strains within *C*. *michiganensis* species and among other *Clavibacter* species.

Comparative genomic analyses revealed that type strain LMG7333^T^ has a genome composition similar to NCPPB382, one chromosome and two plasmids. Overall G + C content and the presence of a PAI region and major virulence genes such as *celA* and *pat-1* were also similar. However, the plasmid size and gene contents were different between the two strains: only 39 and 55% of plasmid DNA of pCM1 and pCM2, respectively, were identical. These findings indicate that high genetic diversity is more present in plasmids than in a chromosome (almost 100% identical between two strains). Moreover, some genes, such as *phpA* and *phpB* genes in pCM2 of NCPPB382, which are *pat-1* paralogs, were missing in pCM2 of type strains LMG7333^T^ (Figure 3), indicating that these two strains might use different virulence factors interaction with host plants.

Among *C*. *michiganensis*, of which their genome sequences are available in the public genome database, the UF1 strain was most similar to *C*. *michiganensis* LMG7333^T^ (Figures 1, 3). Only 4 and 15 genes within the whole genome were specific to the UF1 strain and a type strain LMG7333^T^, respectively (Figure 5). Based on the genome comparison, the UF1 strain might be pathogenic although its pathogenicity has not been reported. Moreover, the genetic diversity between type strain LMG7333^T^ and NCPPB382 was bigger because 91 and 115 genes were specific to each strain, respectively. More analysis of 91 type strain LMG7333^T^-specific genes showed that most of them encode hypothetical proteins, and their homologous are primarily present in bacteria belonging to *Microbacteriaceae* family, including the genus *Clavibacter*. This might indicate that gene exchange or transfer among bacteria in this family occurs.

As expected, the genetic difference among *Clavibacter* species was bigger than that of strains within the single species, *C*. *michiganensis* (Figure 5). The number of core genes among five *Clavibacter* species is 2,345, whereas its number among strains of *C*. *michiganensis* is 2,959. Moreover, in each species, the number of unique genes ranged from 138 to 316. Notably, among *C*. *michiganensis*-specific genes, *tomA* encodes tomatinase. Although the function of these genes is shown to be critical to *C*. *michiganensis* virulence (Kaup et al., 2005), this gene has been defined as a virulence factor in *C*. *michiganensis*. Seipke and Loria (2008) show that *Streptomyces scabies* have tomatinase, and it is related to plant-microbe interactions rather than the pathogenicity of bacterial pathogen, implying that *tomA* in *C*. *michiganensis* might affect the interaction with host plants. There are many genes specific to each *Clavibacter* species (Figure 5B and Supplementary Table 3). However, many of them encode hypothetical proteins with unknown functions. The function of these genes remains determined.

Infection with *C*. *michiganensis* LMG7333^T^ induced typical disease symptoms, canker, and wilting on tomato plants (Figure 6) as observed on tomato plants infected with other pathogenic *C*. *michiganensis*. This means that type strain LMG7333^T^ is also fully pathogenic, and it can be used to understand *C*. *michiganensis*–host plant interactions. Furthermore, the availability of the complete genome information of type strain LMG7333^T^ would make this strain more useful for studies to reveal virulence genes and understand virulence mechanisms against host plants.

## Data Availability Statement

The datasets presented in this study can be found in online repositories. The names of the repository/repositories and accession number(s) can be found in the article/Supplementary Material.

## Author Contributions

E-JO, IP, IH, and C-SO: investigation, writing—review and editing. E-JO, IP, and IH: methodology and formal analysis. C-SO: validation, project administration, and funding acquisition. All authors have read and agreed to the submitted version of the manuscript.

## Conflict of Interest

The authors declare that the research was conducted in the absence of any commercial or financial relationships that could be construed as a potential conflict of interest.

## Publisher’s Note

All claims expressed in this article are solely those of the authors and do not necessarily represent those of their affiliated organizations, or those of the publisher, the editors and the reviewers. Any product that may be evaluated in this article, or claim that may be made by its manufacturer, is not guaranteed or endorsed by the publisher.
