# Supplementary material for: Comparative Genome Analyses of Clavibacter michiganensis Type Strain LMG7333T Reveal Distinct Gene Contents in Plasmids From Other Clavibacter Species
Source: Front Microbiol. 2022 Feb 1;12:793345. doi: 10.3389/fmicb.2021.793345 (PMC8844524; doi:10.3389/fmicb.2021.793345)
Supplement: Supplementary file 1 [file Data_Sheet_1.PDF]

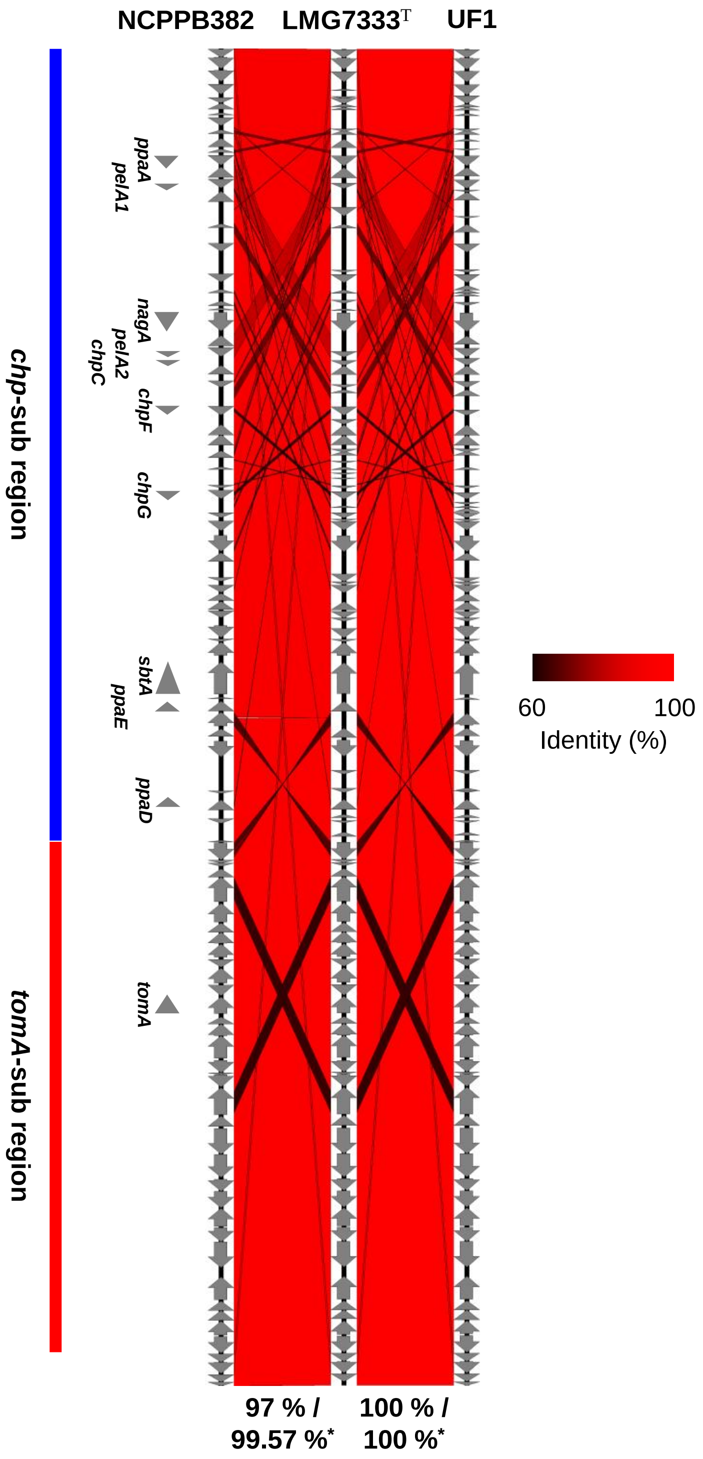

Supplementary Figure 1. Comparison of a pathogenicity islands (PAI) of three *Clavibacter michiganensis* strains. *Percentages indicate represented query cover/identity of *C. michiganensis* LMG7333^{T} PAI with each NCPPB382 and UF1 strain. Identical parts were aligned in red.
